# Implementing Evidence-Based Practices in Hospitals: A Narrative Review of Implementer Roles and Project Attributes

**DOI:** 10.1155/nrp/6463888

**Published:** 2025-10-31

**Authors:** Élise N. Arsenault Knudsen, Kelly Krainak, Morgan Ruona

**Affiliations:** School of Nursing, University of Wisconsin-Madison, Madison, Wisconsin, USA

## Abstract

**Background:**

Evidence-based nursing leads to improved care quality and patient outcomes; however, challenges with implementation prevent that from being achieved. In hospitals, nurses are well positioned across multiple organizational levels to influence the adoption of evidence-based practices (EBP). A better understanding of the roles of those who are serving as implementers of EBP changes in the hospital is needed.

**Purpose:**

This narrative review sought to answer: In hospitals, who (i.e., what roles) are the implementers of nursing-related EBP changes and what are the attributes of those EBP projects? This review aimed to describe (1) the roles and the organizational level of those implementing EBP changes in the hospital for nurses and (2) the attributes of the projects including, its location, the model used to guide the project, and the evaluation measures.

**Methods:**

An iterative, two-phased literature review was conducted. Categorical data were analyzed to achieve the aims.

**Findings:**

Across 158 articles, numerous roles, mostly nursing roles, spanning multiple organizational levels were described. The majority of articles described teams representing multiple levels (*n* = 132; 84%), implementers positioned in unit-level roles (*n* = 37; 23%), and projects occurring at the unit-level (*n* = 127, 80%), using the Joanna Briggs Institute model (*n* = 97, 61%) and an evaluation of process measures (*n* = 77; 49%).

**Conclusion:**

This review identified the implementers of nursing-related practice changes and the attributes of the projects, which provides a rich description of some contextual elements that shape how EBP changes occur in hospitals. This new foundational knowledge can stimulate a systematic approach for improvement and optimization of the implementation process, both in practice and through research. This review highlights the need for tailored and role-specific skills, education, and competencies to equip implementers to lead collaborative and effective teams across hospital systems and maximize the impact of implementation.

## 1. Introduction

The benefits of providing evidence-based care are clear—it improves patient safety, the quality of care, and patient outcomes, while decreasing health care costs [1, 2]. Despite this, the use of evidence-based practice (EBP) remains inconsistent [3, 4], and challenges with the process of implementation and the adoption of EBPs persist [4–6]. The field of implementation science (IS) emerged to discover ways to overcome these challenges and offered numerous strategies to facilitate the adoption of EBPs, but the application of this knowledge to guide implementation in practice settings by nurses is limited [6–8]. Ultimately, the inconsistent use of EBPs impedes its intended goal, which is to achieve optimal patient outcomes [1].

Globally, 29 million nurses have a predominant role in improving patients' health [9], and nurses, as part of their professional standards, are expected to provide evidence-based care [1, 2]. Nurses are well positioned to lead practice changes [10]; their involvement with quality improvement (QI) work has demonstrated positive impacts on patient, nurse, and hospital outcomes [11]. However, the goal of consistently providing evidence-based nursing care remains unrealized [7, 12]. To support nurses' efforts to improve care and facilitate the consistent uptake of EBPs, the process of implementation should be examined and optimized. A key factor to be considered for this examination is the complex, multileveled, and nested structure that shapes the context of providing care in the hospital [10, 13]. Nurses are embedded across multiple organizational levels in hospitals and in a variety of roles, all of which can influence the implementation process. Additionally, non-nurses may also be leading practice changes that influence nursing practice. Yet, the roles leading these EBP changes in nursing practice, and serving as implementers, have not been adequately described. This may hinder implementation; nurses have described an unclear origin or an assumption of a faceless “they” (i.e., administrators) leading nursing-related practice changes as a barrier to implementation [14]. To optimize the implementation of EBPs, we must first identify the implementers of EBP projects in nursing and understand how they are distributed across the multiple organizational levels, which comprises the context of hospitals.

The EBP process is a problem-solving approach, which guides decision-making through the integration of research evidence, clinician expertise, and patient preferences and values [3, 15]. The EBP process is related but distinct from QI, and both are needed to improve care [15]. For example, the EBPs for inserting and maintaining a central venous access device are determined through a literature synthesis, and the adherence to those practices can be monitored through QI processes. However, providing evidence-based care is the standard that nurses are expected to meet and therefore should be the focus of optimization efforts. To guide the EBP process, hospital nurses use multiple models, such as the Iowa model of EBP to promote quality care, the Johns Hopkins model, and the Advancing Research through Close Collaboration (ARCC) model [16]. Positive outcomes, such as improved belief in and knowledge of EBP, have been described as a result of teaching nurses the EBP process [17, 18], and overall, nurses around the world maintain positive beliefs about and have favorable attitudes towards EBP [19, 20]. And yet, a favorable attitude toward EBP does not automatically result in its implementation [19, 20], and the persistent 15- to 17-year gap remains between practices recommended by research evidence and what occurs in routine practice [21, 22].

Education is a heavily relied upon implementation strategy to facilitate the uptake of EBPs. However, in clinical settings, education alone—either about the EBP process or about specific EBPs—does not lead to the consistent implementation and uptake of EBPs in practice [5, 6]. Furthermore, despite educational efforts in both clinical and academic settings, nurses' knowledge about EBP has been insufficient to change practice [20]. The implementation process is complex, involves multiple steps, and requires strategies to overcome individual and organizational barriers [10]. Before additional education or other interventions are created to expand the capacity for implementation, we must have a better understanding of the current state of EBP projects in hospitals, including who is leading the efforts of implementation and the attributes of those EBP projects. This foundational knowledge can inform tailored and specific interventions to optimize the implementation process for hospital nurses.

Therefore, the purpose of this narrative review is to answer the question: In hospitals, who (i.e., what roles) are the implementers of nursing-related EBP changes and what are the attributes of the EBP projects they are leading? A narrative review is well suited to answer this question because it allows for exploring and consolidating the current literature on a wide range of subjects while identifying potential areas for future research [23, 24]. Furthermore, a narrative review allows for categorical analysis, which aligns with the question being asked—rather than a scoping, mapping, or integrative review that would provide a description of the breadth of topical literature or an evidence synthesis focused on improving a specific practice problem, but not answer this question [23, 25]. Specifically, this narrative review aimed to (1) describe the roles and the organizational level of those who are implementing (i.e., the leaders and/or teams) EBP changes in the hospital for nurses and (2) to describe the attributes of projects, including the location of the project (i.e., unit, system), the model used to guide the project, and the types of evaluation measures reported.

## 2. Methods

### 2.1. Literature Search

The authors, in consultation with a health systems librarian, conducted an iterative, two-phased literature search, after ensuring a review on this topic had not already been published. The search was conducted in July and September 2024, using CINAHL and PubMed databases, and was limited to full-text articles published in English between January 2018 and the month of the search (July or September) in 2024.  Table 1 includes the search terms.

**Figure 1:**
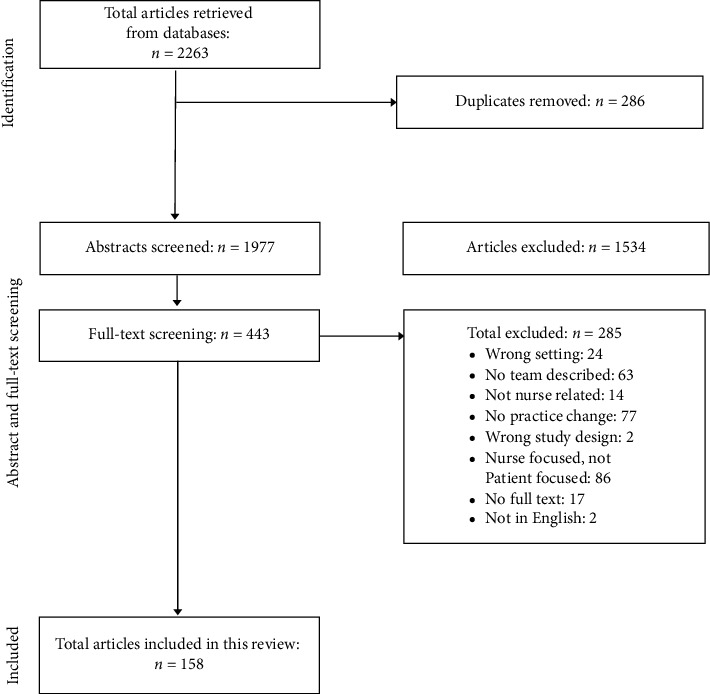
Flow diagram of both phases of literature searches.

Articles were screened to ensure that they met the following criteria: described a practice change in a hospital which impacted nursing practice, the change was focused on improving patient care, and either a team leader or the team was described. The search results and reasons for exclusion are detailed in Figure 1.

Covidence was used to organize the screening, inclusion, and exclusion of the articles. At least two study team members completed the full-text reviews to determine inclusion; exclusion discrepancies were discussed and resolved via consensus. The articles included from the first phase of the literature search revealed a homogenous sample; most of the articles were published in the *Joanna Briggs Institute (JBI) Evidence Implementation* journal using the JBI model. Therefore, a second search was designed in consultation with the librarian to expand the sample. This literature search included “organizational change” and keywords (see Table 1) to intentionally include the three most used EBP models in Magnet designated hospitals, the Iowa Model of EBP, Johns Hopkins EBP Model, and the ARCC model [16]. The details of articles included in each search are included in the Supporting Information (available here).

### 2.2. Data Analysis

Key data elements were extracted from each article included in the review. These key elements were determined a priori, including bibliographic information, purpose, the role of the project leader(s), the organizational level of the leader(s) (i.e., system, department, unit), EBP change team members, if the team members' roles were distributed across multiple organizational levels (yes/no), the organizational level(s) of the practice change (i.e., system, department, unit), the type of evaluation reported (i.e., process measures, patient outcomes), the model used to guide the practice change (i.e., JBI, Iowa), and the country where the EBP change occurred.

To ensure consistency across research team members, all data elements were defined before data extraction began (see Table 2). Data extraction began with each research team member independently extracting data for five articles; the results were compared, and discrepancies were resolved via discussion. This process supported each study team member to consistently apply the key elements' definitions. Independent data extraction continued for the remaining articles, and regular team meetings were held to discuss challenges and address questions. If data elements were unclear to a single reviewer, a second member of the research team reviewed the article to ensure consistent data extraction.

Aligned with the narrative review method and to answer the research questions, the key data elements were analyzed categorically [23]. Apart from the bibliographic information and the purpose statement, each category's frequencies were tallied using Microsoft Excel. During the analysis process, a new question arose across the models used to guide the projects, what similarities exist among the implementers and the project attributes? Therefore, after all data were extracted, a *post hoc* analysis was conducted to examine patterns within and across the models.

## 3. Results

The extracted data from 158 articles were analyzed to describe the implementers and the attributes of the practice change projects.

### 3.1. Implementers of Practice Change

#### 3.1.1. Leaders

Numerous roles were described as the leader or co-leader of the projects, and these roles spanned across multiple organizational levels. Many roles were nursing roles, and those categories included unit nurses (*n* = 27), charge or head nurses (*n* = 17), chief nurses or directors (*n* = 13), advanced practice nurses (*n* = 12; clinical nurse specialist, 7; nurse practitioner, 4; midwife, 1), supervisors or managers (*n* = 8), nurse educators (*n* = 7), clinical nurse consultants (*n* = 5), and specialist nurses (*n* = 4). Faculty or research partners who held positions in academic settings were another role identified as leading practice change (*n* = 20). Additionally, four non-nurses were identified as leaders of the practice change projects.

#### 3.1.2. Teams

Of the 158 articles, 78 (49%) included a description of the project team leader(s)'s organizational level which could be categorized as unit, department, or system. Most often the leader was positioned at the unit-level (*n* = 37; 23%). A team, rather than an individual leader, was described in 43 (27%) of the articles. The full distribution of leaders across organizational levels is further described in Table 3. Based on the description of the project teams, it was noted that 26.5% (*n* = 42) of the articles' projects included a mentor. The professional roles of mentors were diverse, ranging from nurse scientists to EBP experts and improvement staff; it was unclear if all the mentors were nurses. The majority of the project teams included roles that spanned multiple organizational levels (*n* = 132; 84%), but 18 (11%) articles described teams with roles from only one organizational level. In 8 (5%) of the articles, the distribution across organizational levels of the team members could not be determined.

### 3.2. Project Attributes

#### 3.2.1. Project Location

The location of the practice changes predominantly occurred at a unit-level; 127 (80%) articles described a unit-level practice change. Of those, 34 (22%) described the unit-level changes occurring on multiple units (i.e., orthopedic, medical, and oncology units). The remaining articles described practice changes at the department (*n* = 13, 8%) and system level (*n* = 14, 9%); the level was unclear in 1 (0.6%) article. Three (2%) articles described practice changes at multiple organizational levels, one at the unit and department levels and two at the unit and system levels.

#### 3.2.2. Evaluation Metrics

Numerous types of metrics and often a combination of metrics were used to report the impact of the practice change in each sampled project. The categories of these evaluation metrics are described in Table 2. Among the 158 articles, 77 (49%) reported solely on process measures and 24 (15%) solely reported on patient outcomes. About a third (*n* = 57; 36%) of the articles reported on multiple types of evaluation metrics. Of these, 40 (70%) articles reported on both patient and process outcomes, 6 (4%) reported on patient and nurse outcomes, 5 (9%) reported on patient and system outcomes, and 3 (8%) reported on nurse and process measures. The remaining 3 (5%) articles reported on a combination of process, nurse, and patient metrics.

#### 3.2.3. Model Used to Guide Project

Most authors described the model that was used to guide the practice change. Of the 158 articles, 97 (61%) described projects guided by the JBI model, 23 (15%) were guided by the Iowa Model, 6 (4%) used PDCA or PDSA, and 1 (0.6%) of the articles used the Johns Hopkins model. Eight (5%) articles used other models, including the i-PARIHS, Melnyk, and Fineout-Overholt's model for EBP, the continuous QI model of the Fudan University Evidence-Based Nursing Center, Knowledge-To-Action, Participatory Action Research method, or complexity theory to guide the practice change. Nineteen (12%) articles did not report a model, and 4 (3%) used multiple models.

During the post hoc analysis, a comparison of project attributes across models was conducted. The results are presented in Table 4. In summary, of the 97 articles reporting projects guided by the JBI model, 29 (30%) described a unit-level project leader and 83 (86%) unit-level practice changes. Furthermore, these projects predominantly reported process measures (*n* = 70, 72%), and 29 (30%) of projects indicated that a mentor was part of the project team. For comparison, the summary of project attributes guided by the Iowa Model indicates a team, rather than a single project leader, led the projects in 10 (43%) of the articles and 6 (26%) of the teams included a mentor. Most of the project reported on patient outcomes (*n* = 10, 40%) or a combination of measures (*n* = 9, 39%) and described unit-level practice changes (*n* = 19; 83%).

#### 3.2.4. Setting

This sample of articles described practice changes occurring in hospitals located in the United States and Canada (*n* = 58, 37%), China (*n* = 31, 20%), Singapore and Taiwan (*n* = 19, 12%), Spain and Portugal (*n* = 11, 7%), Brazil (*n* = 9, 6%), the Czech Republic, Poland, Romania, and Switzerland (*n* = 9, 6%), Australia (*n* = 7, 4%), Iran (*n* = 4, 3%), the United Kingdom and Ireland (*n* = 3, 2%), Ethiopia and Malawi (*n* = 2, 1%), and India (*n* = 1, 0.6%). Four (3%) articles did not list a country or region.

## 4. Discussion

This narrative review described the roles of those serving as implementers of EBP changes within the hospital for nurses. The diversity of roles is positive; the inclusion of academic partners in this work aligns with current initiatives focused on building capacity for using IS in practice [26], and the involvement of unit nurses could facilitate changing practice [14]. Additionally, by describing the distribution of the implementers across the hospitals' organizational structure and the attributes of the EBP projects, this review provides valuable insights into the context and structure of practice changes in hospitals and contributes new knowledge to the field of IS. The number of articles published describing practice changes in hospitals since 2018 demonstrate that nurses are actively working to improve their practice using EBP, suggesting that nurses are responding to the call of providing evidence-based care. Simultaneously, the findings of this review provide additional insight into where gaps may lie between these projects' efforts and the goal of consistently providing evidence-based care, revealing practical implications for nurses in practice and for nursing research.

The identification of multiple and diverse roles serving as implementers across hospital organizations provides specificity about which nursing roles may reap the greatest benefit of possessing strong skills, knowledge and competence in EBP, and implementation. These attributes have been described as essential to improve nurses' readiness for EBP, to overcome barriers (i.e., lack of education, low EBP competence) and to actualize EBPs being used in practice [19, 20, 27]. The range of roles identified in this review as leading or coleading practice changes, from academic partners to unit and charge nurses to chief nurses, highlights the need for defining the nuanced skills needed and tailored education required to ensure each role can effectively and successfully lead implementation efforts. Defining and evaluating these role-specific interventions could occur both pragmatically in practice and systematically through research.

This review found that about a quarter of projects did not identify a singular leader of the EBP project but rather described the leadership distributed among multiple team members and, overwhelmingly, these team members spanned multiple levels of the organization. These findings contrast with the current literature that primarily highlights the role of the nurse manager when describing leaders of EBP [12, 28, 29]. The integration of varied roles across organizational levels on project teams reinforces the notion that nurses are influencing the implementation process at each level in the organization; however, this multilevel team complexity has not been adequately explored in IS. Additionally, adjusting or adapting implementation leadership behaviors associated with successful implementation [12] based on roles or position in the organizational hierarchy may be warranted. Further examination of this distribution of implementation efforts across varied roles is needed to understand the strengths and areas for optimization.

Overall, teamwork has been minimally examined within the context of implementation [30, 31], and yet, forming a team is a key step in the EBP process outlined in models, like the Iowa Model and JBI [32, 33]. The articles in this review described teams working on the projects; however, despite team members spanning multiple organizational levels, most articles reported unit-level leaders leading unit-level practice changes. This highlights the need for role-specific, tailored guidance for not only how these interorganizational roles can facilitate the uptake of EBPs but also spread them beyond the unit-level, such as to the department or hospital level [5]. The addition of mentors to the team is one evidence-based strategy to facilitate and promote the uptake of EBPs [34], and the responsibilities of the various steps in the EBP process could be divided between the unit-based nurses and their mentors [20]. However, this review reinforces that challenges exist to include mentors, as only a quarter of the sampled articles described mentors as part of the team; limited access to mentors in practice is an often-cited barrier to EBP [19]. Additional resources and support are likely needed in hospitals to increase the number and availability of mentors to join teams and support these projects. Additional research is needed to establish recommendations to guide teams on how to optimize their roles, detailing taskwork for individuals, and the collective teamwork [35] is needed to maximize their impact on the implementation process.

The impact of implementing EBPs is essential to capture. The choice of evaluation metrics should help to underscore the relationship between the implementation of EBPs and its impact on the quality of care and patient safety. Nurse executives use that information to make informed decisions about priority setting and the related budget allocations [3, 22] which influences the structures and resources available to support EBPs and practice change. Moreover, when nurses know about the positive impact on patient outcomes from the practice change, it can facilitate the implementation and sustainment of a new practice [14, 36]. However, in this review, about half of the articles reported on process measures, rather than patient outcomes. While this finding is not unique, it aligns with the literature search conducted by Connor et al. [1] for a systematic review, and it should be examined more closely to understand how the processes put in place with these projects have met the goal of EBP—achieving high-quality patient outcomes [3].

The low number of EBP projects evaluating patient outcomes in this review may reveal additional insight about organizational-level barriers and challenges teams face when implementing an EBP change and selecting evaluation measures. For example, our *post hoc* analysis suggests a link between the model used to guide the project and the selection of evaluation measures. Each model provides slightly different guidance on evaluation; for example, the JBI model includes a step of auditing the practice of interest [33], which often is completed by auditing documentation of the care that was provided. By comparison, the Iowa model recommends creating an evaluation plan [32], which may prompt broader thinking and decision-making about the types of outcomes measured. Interestingly, in this review, each of the articles that described using the PDCA/PDSA model reported on patient outcomes or a combination of patient and process metrics. This is a bit surprising because this is a QI model focused on improving processes, more so than patient outcomes [15]. The predominance of using process measures raises the question of how well the impact of these projects is understood and if the goal of improving patient outcomes is actually being met. Further exploration of how implementers select evaluation metrics and an understanding of underlying barriers and facilitators to the evaluation steps in the implementation process are needed. For example, a greater understanding of the resources and skills required for project leaders to easily obtain, analyze, and report on data from various sources (i.e., electronic health record, quality data) to comprehensively describe the impact of implementation on care quality, patient safety, and patient outcomes would be beneficial. While the measurement of outcomes of IS studies has been delineated [37], additional guidance may be needed for implementers in practice settings for how to best evaluate the impact of practice changes. Increased awareness and guidance for using EBP evaluation frameworks, such as knowledge, attitudes, behaviors, outcomes, and balancing measures (KABOB), in conjunction with EBP models could optimize the implementation process from planning through evaluation and sustainment [5]. Additional focus on how to support comprehensive evaluation of EBP projects may better inform implementation efforts and help to ensure that both EBPs are being adopted and the gaps in patient outcomes are closed.

### 4.1. Limitations

By design, this review included a representative sample of projects, not an exhaustive sample. The inclusion and exclusion criteria narrowed the search to those articles describing recent practice changes published in two prominent biomedical and nursing databases and accommodated the study team's language capabilities. While these criteria limited the search, it still provided access to a substantial number of citations and resulted in an adequate representative sample. Yet it is possible that an analysis of a different sample of articles could lead to different results. However, this review was strengthened by including publications that reported a change in practice alongside the attributes of the projects. This approach contributes valuable new knowledge by describing what is occurring in hospitals around the world. That being said, this review only included full-text publications, which may not capture the breadth of projects hospital nurses are engaged in; practicing nurses may be more likely to disseminate within their organizations or at professional conferences, rather than through publications in academic journals. However, the few conference abstracts discovered while refining the literature search did not include enough detail for meaningful data extraction.

To overcome the known limitations of a narrative review method, namely, the lack of transparency of the literature search and data analysis [23, 24], the authors intentionally included this information to strengthen this review. As described, the search terms of this review were focused on EBP and implementation projects, but the terms and methods used by authors to describe their work varied across publications. The authors ensured that each article met the inclusions criteria. The quality of the projects was not assessed, nor was the language used to describe the projects critiqued—it was not the goal of the review; however, the variation likely shapes the findings. The variation in language likely reflects the enduring confusion between research, QI, and EBP [15], rather than the quality of projects conducted.

## 5. Conclusion

In nursing, we should prepare the context of our practice settings to facilitate the implementation of EBPs [10]. One important step in that preparation is to understand the current landscape of work being done in hospitals by nurses. This review identified the implementers of nursing-related practice changes and the attributes of the projects, which provides a rich description of some of the contextual elements that shape how EBP changes occur in hospitals. This new foundational knowledge can launch us towards a more systematic approach for improvement and optimization of the implementation process, both in practice and through research. Additionally, the findings of this review highlight the need for tailored, role-specific skills, education, and competencies to equip implementers to lead collaborative and effective teams across hospital systems and maximize the impact of implementation. Future work should identify strategies to leverage these nursing roles to optimize the implementation process to facilitate the uptake of EBPs and improve patient outcomes.

## Figures and Tables

**Table 1:** Literature search: keywords and databases.

Date of search	Databases searched	Keywords^∗^
July 2024	CINAHL	(nurse OR nurses OR nursing) AND (hospital OR acute OR inpatient OR ward) AND (“best practice implementation project” OR “evidence-based practice project”)
	PubMed	(nurse OR nurses OR nursing) AND (acute OR inpatient OR ward) AND (“implementation project” OR “best practice implementation project” OR “evidence-based practice project”)
September 2024	PubMed and CINAHL	(nurse OR nurses OR nursing) AND (hospital OR acute OR inpatient OR ward) and (Iowa Model OR “The Iowa Model of Evidence-Based Practice to Promote Quality Care”)
	PubMed and CINAHL	(nurse OR nurses OR nursing) AND (hospital OR acute OR inpatient OR ward) AND (“Johns Hopkins Nursing Evidence-based Practice Model” OR Johns Hopkins Model)
	PubMed and CINAHL	(nurse OR nurses OR nursing) AND (hospital OR acute OR inpatient OR ward) AND (“advancing research and clinical practice through close collaboration” OR ARCC)
	PubMed	(nurse OR nurses OR nursing) AND (hospital OR acute OR inpatient OR ward) AND ((change, organizational [MeSH terms]) OR (organizational change))
	CINAHL	(nurse OR nurses OR nursing) AND (hospital OR acute OR inpatient OR ward) AND organizational change

^∗^Of note PubMed searches MeSH terms with keyword searches automatically.

**Table 2:** Key data elements defined for data extraction.

Data element	Definition
Leader role	The title of the leader's role used by the author(s)
Leader's organizational level	The leader's organizational level was assigned a category in alignment with the author's language or description of the role, with responsibilities:
System	• Across the whole hospital or health care system (i.e., CNS for specific population across hospital; nurse scientist for system)
Department	• Across multiple units together or based on the author's language (i.e. department of orthopedics)
Unit	• On a single unit or is a local leader (i.e., nurse manager, charge nurse, unit nurse)
Academic partners	• As part of a university or academic institution
Team members	The team composition was copied from each article
Team member span organizational levels	Based on the description of the team members, using the definitions above; dichotomized to yes or no
Organizational level of practice change	The project's organizational level where the practice change occurred was categorized based on the description with deference to the author's description of where the implementation occurred:
System	• Across the whole hospital
Department	• Across multiple units that are cohesively related
Unit	• On a single unit
Multiple units	• On multiple different units that are not described as a department (i.e., medical unit & ED)
Evaluation metrics	The metrics for evaluating the practice change measure:
Process measures	• The steps in the process to carry out the practice (i.e., rate of documentation of screening; change in knowledge)
Patient outcome	• The patient impact of the new practice (i.e., falls rate; length of stay; readmission rates; patient satisfaction)
Nurse outcome	• The impact of the practice change on nurses (i.e., nurse retention; confidence in care)
System outcome	• The impact on the health care system (i.e., cost avoidance; cost savings)
Model used	The model (s) indicated by the author(s)
Country	The country where the project occurred, as indicated by the author(s)

**Table 3:** Roles leading practice change projects and corresponding organizational level.

Organizational level of leader	Distribution across articles frequency (%)	Roles leading practice projects (number of leaders described in that role)
System	9 (6%)	Director of nursing/care (4) (1 article reported 2 system-level coleads across director and chief nurse roles)
		Chief nurse (2): education and quality (1), chief nursing officer (1)
		Clinical nurse consultant (1+) (1 article reported multiple Clinical Nurse Consultants as co-leaders)
		Sepsis coordinator (1)
		Educator (1)

Department	10 (6%)	Department director/supervisor (3)
		Head/senior nurse (4)
		Clinical nurse specialist (CNS) (1)
		Nurse clinician (1)
		Note: 1 article indicates the department led the project, but a specific role was not described

Unit	37 (23%)	APNs (7): nurse practitioner (3), midwife (1), CNS (2)
		Unit manager/supervisor (3)
		Head/charge nurse (9) (3 head nurses coled 1 project)
		Unit nurse (20) (2 unit nurses coled 2 projects, and 3 nurses coled 1 project)
		Nurse champions (exact number not reported; 1 article)
		Nurse residents (exact number not reported; 1 article)
		Unit Educator (4) (2 unit educators coled 1 project)

Academic Partners	11 (7%)	Professor/faculty/teacher/researcher (9) (2 faculty members coled 1 project)
		PhD student (1)
		Academic staff (2)

Co-leaders span organizational levels	11 (7%)	Unit nurse (3) (2 unit nurses & 1 CNS coled 1 project)
		CNS (3) (2 unit nurses & 1 CNS coled 1 project)
		Head nurse (1)
		Nurse educator (2)
		Head nurse of department/service (2)
		Head of hospital (1)
		Non-nurses (3): MD (1), clinical pharmacist (2)
		Director of nursing (1)
		Chief nurse of quality (1)
		Faculty researcher (4)
		Supervisor/manager (3)
		Clinical nurse consultant (1)

Leader role unclear (i.e., leader is alluded to, but role is unclear)	22 (14%)	1 article described the project as APRN-led without providing any other details

Level of leader unclear	15 (10%)	Unit nurse (3) (2 unit nurses coled 1 project)
		Clinical dietitian (1)
		Nurse practitioner (1)
		Director of care (1)
		Supervisor nurse (2) (2 supervisor nurses coled 1 project)
		Head nurse (1)
		Researchers/research nurse (4+) (2 scientific nurses coled project)
		Nurse champions (5) (5 nurse champions coled 1 project)
		Consultants (4): Clinical nurse consultant (3) (3 clinical nurse consultants coled 1 project), mental health consultant (1)
		RN lean coach (1)

Team described (no team leader indicated)	43 (27%)	Not applicable

**Table 4:** Leaders' and projects' organizational levels and evaluation metrics.

	Model used to guide project						
	JBI	Iowa model	PDSA/PDCA	John Hopkins	Other	No model	Total
	*n* = 97 (62%)	*n* = 23 (15%)	*n* = 6 (4%)	*n* = 1 (0.6%)	*n* = 8 (5%)	*n* = 19 (12%)	*N* = 154^∗^
*Organizational level of leader n (% of articles/model)*							
System	5 (5%)	1 (4%)	0 (0%)	0 (0%)	0 (0%)	2 (11%)	8
Department	8 (8%)	1 (4%)	1 (17%)	0 (0%)	0 (0%)	0 (0%)	10
Unit	29 (30%)	5 (22%)	0 (0%)	0 (0%)	0 (0%)	2 (11%)	36
Co-leaders spanned levels	9 (9%)	0 (0%)	0 (0%)	0 (0%)	1 (13%)	0 (0%)	10
Level of leader is unclear	23 (24%)	6 (26%)	1 (17%)	0 (0%)	2 (25%)	5 (26%)	37
Team described, no leader described	12 (12%)	10 (43%)	4 (67%)	1 (100%)	5 (63%)	10 (53%)	42
Academic partners	11 (11%)	0 (0%)	0 (0%)	0 (0%)	0 (0%)	0 (0%)	11
Mentored projects	29 (30%)	6 (26%)	2 (33%)	1 (100%)	1 (13%)	2 (11%)	41^∗^

*Organizational level of project n (% of articles/model)*							
System	2 (2%)	2 (9%)	1 (17%)	0 (0%)	2 (25%)	6 (32%)	13
Department	11 (11%)	0 (0%)	1 (17%)	0 (0%)	0 (0%)	1 (5%)	13
Unit	83 (86%)	19 (83%)	4 (67%)	0 (0%)	6 (75%)	12 (63%)	124
Multiple Units	23 (28%)	4 (21%)	2 (50%)	0 (0%)	2 (33%)	2 (17%)	33
Multiple levels	1 (1%) (unit & system)	1 (4%) (unit & department)	0 (0%)	1 (100%) (unit & system)	0 (0%)	0 (0%)	3
Level unclear	0 (0%)	1 (4%)	0 (0%)	0 (0%)	0 (0%)	0 (0%)	1

*Evaluation metrics reported n (% of articles/model)*							
System outcomes	0 (0%)	0 (0%)	0 (0%)	0 (0%)	0 (0%)	0 (0%)	0
Patient outcomes	3 (3%)	10 (43%)	3 (50%)	0 (0%)	1 (13%)	7 (37%)	24
Process measures	70 (72%)	4 (17%)	0 (0%)	0 (0%)	1 (13%)	2 (11%)	77
Multiple evaluation types:	24 (25%)	9 (39%)	3 (50%)	1 (100%)	6 (75%)	10 (53%)	53
Patient & Process	24 (100%)	5 (56%)	3 (100%)	1 (100%)	2 (33%)	4 (40%)	39
Patient & Nurse	0 (0%)	2 (22%)	0 (0%)	0 (0%)	1 (17%)	1 (10%)	4
Patient & System	0 (0%)	1 (11%)	0 (0%)	0 (0%)	1 (17%)	2 (20%)	4
Nurse & Process	0 (0%)	1 (11%)	0 (0%)	0 (0%)	1 (17%)	1 (10%)	3
Process, Nurse & Patient	0 (0%)	0 (0%)	0 (0%)	0 (0%)	1 (17%)	2 (20%)	3

Abbreviations: JBI = Joanna Briggs Institute, PDSA/PDCA = Plan, Do, Study/Check, Act.

^∗^4 articles used multiple models and were not included in this post hoc analysis; therefore, they are not included in this table. One of these articles included a mentored project.

## Data Availability

The data that support the findings of this study are available from the corresponding author upon reasonable request.

## Supporting Information

Literature Search Results per Model.

This table provides additional detail about the number of articles found in each phase of the literature search and the tally of the included articles per model.
