# Supplementary material for: Implementing Evidence-Based Practices in Hospitals: A Narrative Review of Implementer Roles and Project Attributes
Source: Nurs Res Pract. 2025 Oct 31;2025:6463888. doi: 10.1155/nrp/6463888 (PMC12595238; doi:10.1155/nrp/6463888)
Supplement: Supporting Information — Additional supporting information can be found online in the Supporting Information section. [file 6463888.f1.docx]

**Supplementary Table**

| *Literature Search Results per Model* | | | | | | | | |
| --- | --- | --- | --- | --- | --- | --- | --- | --- |
|  | Search #1 | Search #2 | | | | | |  |
|  |  | Iowa | Johns Hopkins | ARCC | | Organizational Change | |  |
| Studies Retrieved | 231 | 309 | 69 | 28 | | 1626 | |  |
| Articles Removed/Excluded | 114 | 294 | 69 | 28 | | 1600 | |  |
| Number of articles included | 117 | 15 | 0 | 0 | | 26 | |  |
|  |  |  | | | | | |  |
| Model | Search #1  (of 117 included articles) | Search #2  (of 41 included articles) | |  | |  | |  |
|  |  |  |  |  | |  | |  |
| Iowa Model | 8 | 15 | |  | |  | |  |
| Iowa with Johns Hopkins | 1 | 0 | |  | |  | |  |
| Iowa with Kotter’s and PDSA | 0 | 1 | |  | |  | |  |
| JBI | 95 | 2 | |  | |  | |  |
| Johns Hopkins | 1 | 0 | |  | |  | |  |
| ARCC | 0 | 0 | |  | |  | |  |
| PDCA/PDSA | 1 | 5 | | |  | |  | |
| No model | 6 | 13 | | |  | |  | |
| Other | 5 | 5 | | |  | |  | |

*JBI = Joanna Briggs Institute*; *ARCC = Advancing Research and Clinical practice through Close Collaboration*; *PDCA/PSA = Plan-Do-Check/Study-Act*
